# A Hybrid Ultra-Wideband and Inertial Measurement Unit Sensor System Can Accurately Measure the Sprinting Performance in Young Male Soccer Players

**DOI:** 10.3390/jfmk11040421

**Published:** 2026-10-14

**Authors:** Vassilios Panoutsakopoulos, Panagiotis Kitsikoudis, Anestis Damianou

**Affiliations:** Biomechanics Laboratory, School of Physical Education and Sport Science at Thessaloniki, Aristotle University of Thessaloniki, 54124 Thessaloniki, Greece; pkitsik@phed.auth.gr (P.K.); adamiang@phed.auth.gr (A.D.)

**Keywords:** biomechanical analysis, sport performance, soccer, ultra-wideband technology, force–velocity profile, power, acceleration, validity, sprint, youth sports

## Abstract

**Background**: This study investigated the concurrent validity and agreement of a hybrid ultra-wideband (UWB) and inertial measurement unit (IMU) wearable sensor system (K-Power) against criterion photocell timing gates during a 30 m sprint test, while secondarily exploring associations between 30 m sprint time (t30) and horizontal force–velocity (F-V) profile parameters in youth soccer players. **Methods**: Eighteen semi-professional male U19 soccer players executed three maximal 30 m sprints on an outdoor grass field. Sprint split times (0–10 m, 10–20 m, 20–30 m, and 0–30 m) were recorded simultaneously using the K-Power system (200 Hz) and four pairs of electronic timing gates. Mechanical F-V parameters—maximal theoretical force (F_0_), maximal theoretical velocity (V_0_), and maximal mechanical power (P_MAX_)—were derived from the sensor data. Concurrent validity was assessed using bias, limits of agreement (LoA), Pearson’s correlation, and intraclass correlation coefficients (ICC{A,1}). Relationships among parameters were evaluated using Kendall’s correlation. **Results**: The K-Power system demonstrated excellent absolute agreement for total sprint time (t30; ICC = 0.967; mean bias = –0.011 s) and strong correlations across all split intervals (*r* = 0.921 to 0.974). No proportional bias was observed. Regarding the F-V profile, t30 exhibited moderate negative correlation with P_MAX_ (*τ_b_* = −0.428, *p* = 0.015). **Conclusions**: The hybrid UWB-IMU sensor system is a valid and practical field-based tool for monitoring linear sprint performance and mechanical capabilities in soccer. Furthermore, sprint performance in youth soccer players over 30 m is primarily associated with maximal power capabilities during the initial steps rather than V_0_.

## 1. Introduction

The accurate monitoring of sprint acceleration, maximal velocity, and power output capabilities is of concern in team sports, with sprinting skills being suggested to be of high importance in soccer [1,2], while practitioners seek for valid, user-friendly, field-deployable sensors to allow frequent monitoring and assessment of sprint performance and its biomechanical parameters [3]. Lately, it has been supported that frequent, low-burden field monitoring solutions are essential for soccer coaching staff and practitioners, because modern competition schedules are highly volatile, causing rapid and uneven shifts in player workload that cannot be managed with standard weekly routines [3].

The biomechanical determinants of soccer players’ sprinting ability are often evaluated through the force–velocity (F-V) profile, which characterizes a player’s neuromuscular capability to apply horizontal force to the ground across different running speeds [4]. Specifically, maximal theoretical force (F_0_) reflects the ability to generate high horizontal ground reaction forces during the initial explosive steps of acceleration, while maximal theoretical velocity (V_0_) represents the athlete’s top-end speed capability [4,5]. Typically, photocell split times acquired by photocell gates placed 5 m to 10 m apart [6] are the practical input for field-based horizontal F-V profiling by providing the time-distance data used to reconstruct the sprint’s velocity–time relationship [4].

Past research suggests that F_0_, V_0_, and maximal mechanical power (P_MAX_) are strongly related to acceleration, while differences were found to exist due to playing position, competitive level, and biological maturation status [7]. Furthermore, not all F-V profile parameters demonstrated strong predictive relationships with sprint performance. V_0_ and F_0_ were found to exhibit trivial to small relationships with sprint performance [8]. Thus, based on the profile of the participants, the relationship between F-V profile parameters and sprint performance differs considerably across studies and measurement contexts.

The biomechanical requirements of sprinting change drastically in the transition from the early acceleration to the maximum sprinting speed phase [9,10]. Accurately capturing these distinct phases in field settings requires precise split times (e.g., 5 m or 10 m intervals), as the distance and timing-gate configuration directly influence the accuracy of the speed profile and the interpretation of the athlete’s mechanical capabilities [11]. Field-based speed measurements using electronic timing gates (photocells) are widely considered [12] as the “gold-standard” method for overall and/or split time evaluation despite the limited accuracy in short sprints [13], whereas radar and laser guns are used for instantaneous velocity profiling. Furthermore, a recent study confirmed that Global Positioning System (GPS) devices are reliable for measuring maximum speed and split times during both linear and curvilinear sprint tests [14]. Nevertheless, these devices present distinct logistical limitations and disadvantages, i.e., the time spent setting up the devices, the lack of measuring continuous data at large sampling rates, etc. [15]. In terms of measurement validity and reliability, GPS demonstrated the highest validity and reliability when compared to reference radar systems, while smartphone applications, despite the reported high intra- and inter-rater reliability, exhibit reduced validity compared to GPS and radar devices [16].

The K-Power system (KINVENT Biomécanique, Montpellier, France) could resolve the distinct logistical limitations of other measurement methods, since it is a wearable, wireless motion-tracking device engineered for sports performance analysis and clinical rehabilitation assessment [17]. By integrating nine-degree-of-freedom inertial measurement unit (IMU) with ultra-wideband (UWB) ranging, the system delivers high-precision kinematic data such as distance, velocity, and acceleration during dynamic, explosive tasks (i.e., jumps, sprints). Operating both indoors and outdoors, K-Power utilizes a hybrid sensing framework where the IMU captures high-frequency motion dynamics, while the UWB subsystem establishes absolute distance references via real-time communication with fixed anchors. To maintain tracking integrity under fast motion or indoor reflections, a dedicated signal-processing pipeline automatically selects the most stable measurement path, filters raw data, and rejects multipath interference. Finally, a proprietary sensor fusion algorithm merges the short-term responsiveness of the IMU with the long-term spatial stability of UWB ranging. This eliminates sensor drift and reconstructs a continuous, accurate velocity and position profile. Nevertheless, research [17] has established the concurrent validity of this system for sprint performance over an entire distance, but not for separate splits within a sprinting test.

While extensive research has profiled the macroscopic F-V characteristics of athletes, the specific interactions between performance in total sprint time (0–30 m) and the mechanical F-V profile parameters remain under-investigated in youth soccer players. Thus, the objectives of this exploratory study were to investigate the concurrent validity and agreement of the measures acquired from the K-Power sensor system against criterion timing gates for recording each 10 m split in a 30 m sprint test in young soccer players, as well as to examine the relationship of sprint performance with the F-V profile parameters. Based on previous research [17], it was hypothesized that the K-Power sensor system would yield valid results against the criterion measure of photocell gates, while total sprint time will be related with the F-V profile parameters, namely P_MAX_ and maximum sprint velocity (V_MAX_), given the acceleration-dominant nature of a 30 m sprint.

## 2. Materials and Methods

### 2.1. Design of the Study

A convenient sample of male youth soccer players was examined in the middle of the competitive season to evaluate the comparative validity and agreement of performance in a maximal 30 m sprint test recorded by the K-Power sensor system compared to gold-standard criterion measures (four pairs of photocell timing gates placed to measure three 10 m splits) during a single testing session. The acquired data were then further analyzed to calculate the parameters that depict the F-V profile.

### 2.2. Participants

Eighteen (*n* = 18) male semi-professional U19 soccer players (age: 17.7 ± 0.5 yrs, body height: 1.79 ± 0.08 m, body mass: 70.5 ± 8.2 kg) participated in the study. Because recruitment relied on a convenience sample based on team availability, a sensitivity analysis was conducted *post hoc* using the G*Power v.3.1.9.7 software [18] to determine the detectable effect size. This analysis revealed that the available sample size of 18 players provided a statistical power of ≈0.77 at a significance level of *a* = 0.05 to detect strong correlations (*r* ≥ 0.60) for the primary outcome.

The inclusion criteria were the absence of disabilities and other health-related disorders, systematic participation in five or more training sessions per week that included sprint-specific exercises, competitive experience for at least five years, and the avoidance of performing strenuous activity for a 24 h period before the testing session. The exclusion criterion was the occurrence of injury within three months before the measurements.

Signed informed consent forms and (were appropriate) written parental/guardian consent forms were collected before participation in the measurements. Ethical approval was granted by the Institutional Research Ethics Committee (Approval Number: 268/2025).

### 2.3. Experimental Procedure

At first, the body height was measured with a Seca 220 stadiometer (Seca Deutschland, Hamburg, Germany), while the body mass was measured using a pair of K-Plates force plates (KINVENT Biomécanique, Montpellier, France). After the measurement of the anthropometric parameters, the examined players performed their standardized warm-up protocol that comprised 6 min of running at moderate intensity (60% of their maximum heart rate), followed by a 5 min session that included dynamic stretching exercises. The warm-up was concluded with a series of progressively faster and longer sprints, following their usual practice.

The sprint test was executed outdoors on a common grass soccer field. The players wore their soccer shoes and their kit. A familiarization session was conducted first to adjust the sensor on the lower back of the participants, to practice the sprint distance, and to get accustomed with the preferred starting stance in relation to the experimental setup. The starting stance was a self-preferred 2-point (split-stance) stance, where the toes of the lead foot were just behind the 0.5 m mark before the start line [19]. Afterwards, participants performed three self-initiated maximal 30 m sprint tests, with a passive inter-trial rest of 3 min as in typical protocols in soccer sprint research [16].

Data were recorded with the K-Power system (KINVENT Biomécanique, Montpellier, France; sampling frequency: 200 Hz). The first sensor of the system was fixed firmly with a specialized belt on the athlete’s lumbar area. The second sensor (“anchor”) was fixed on a stable tripod (height: 0.9 m) that was placed 3 m behind the start line. After the establishment of inter-sensor communication, participants were allowed to start the sprint test. According to previous research [17], a velocity threshold of 0.2 m/s and a covered distance of 0.5 m were defined as the start criterion for the K-Power system.

The recorded data were transmitted through low-energy Bluetooth signal to a Samsung Galaxy A04s smartphone (Samsung Electronics Co., Ltd., Suwon, Republic of Korea), where the KINVENT v.2.23.0.5 (KINVENT Biomécanique, Montpellier, France) mobile application was installed. With the use of the application, the overall sprint performance (t30), the 10 m and 20 m time splits (t10 and t20, respectively), V_MAX_, and the distance from the start line at which V_MAX_ was achieved were extracted (SV_MAX_). In addition, according to the biomechanical model proposed by Samozino et al. [20], the F-V profile parameters were computed using a custom analytical routine in R software (version 4.5.3, R Foundation for Statistical Computing, Vienna, Austria). The input variables for this calculation included the raw time-distance data, alongside the athlete’s body mass and stature. Assuming that the player’s center of mass forward velocity follows a mono-exponential function during the sprint acceleration phase, instantaneous horizontal velocity and acceleration were derived. Net horizontal ground reaction force was then modeled by adding aerodynamic friction to the product of body mass and forward acceleration. The linear relationship between horizontal force and velocity yielded the following extrapolated parameters: maximal theoretical force (F_0_, namely the y-intercept), maximal theoretical velocity (V_0_, namely the x-intercept), maximal mechanical power (P_MAX_, calculated as F_0_ × V_0_ / 4), the slope of the F-V profile (FV_SLOPE_), and the decrease in ratio of force (DRF).

Simultaneously, t30, as well as each 10 m time splits, were measured with four pairs of timing gates (WITTY System, Microgate, Bolzano, Italy). The gates were positioned as follows: at 0.5 m before the starting line, at the 10 m mark, the 20 m mark and at the finish line (namely the 30 m mark). The photocells were placed on fixed tripods and adjusted, as recommended by Yeadon et al. [21] at approximately hip height (~1 m from the surface of the grass). The acquired data from the timing gates were used as the criterion system.

### 2.4. Statistical Analysis

The fastest 30 m sprint trial recorded by the criterion system and the data acquired by the test system from the same trial were selected for further analysis. Statistical analysis initially involved calculating the mean absolute error (MAE) as the absolute difference between photocell and K-Power data, alongside the root mean square error (RMSE) and mean absolute percentage error (MAPE) to quantify the magnitude and relative percentage of the measurement error. The primary outcomes were Bland–Altman agreement (bias and limits of agreement; LoA) and intraclass correlation coefficient (ICC) for absolute agreement. Systematic bias was subsequently evaluated using a paired-samples *t*-test to determine the mean difference between devices. To quantify relative agreement, a two-way mixed-effects ICC (ICC{A,1}) for absolute agreement was utilized and interpreted as >0.90 (excellent), 0.75–0.90 (good), 0.50–0.74 (moderate), and <0.50 (poor) [22]. Furthermore, Pearson’s correlation coefficient *r* was used to evaluate the linear relationship, while the concordance correlation coefficient (CCC) measured overall concordance. Absolute agreement was visually assessed using Bland–Altman plots to establish 95% LoA, and proportional bias was tested by regressing the signed differences against the mean values of the paired measurements. Finally, a sensitivity analysis was conducted by systematically removing individual player data to confirm that the bias and agreement findings were robust and not disproportionately driven by individual outliers.

Measurement error was quantified through several distinct metrics. Typical error (TE; the standard deviation differences ratio to the square root of 2) was calculated to establish the standard error between devices, while the standard error of measurement (SEM) was derived from the criterion measure to assess precision. The SEM was then used to calculate the minimal detectable change (MDC) at a 95% confidence level (MDC95 = SEM × 1.96 × √2), identifying the smallest change required to reflect a true performance variation beyond random error. Relative absolute error was captured via the coefficient of variation (CV; [TE/criterion group mean] × 100), with thresholds set at: <5% (good), 5–10% (moderate), and >10% (poor). For practical application within this cohort, which lacked a specific physiological performance anchor, the smallest worthwhile change (SWC) was established as 0.2 times the between-subject standard deviation of the criterion measure. This distribution-based approach represents the smallest standardized effect size (Cohen’s *d* = 0.2) capable of altering an athlete’s rank within the population [23]. Finally, Cohen’s *d* effect sizes for paired comparisons quantified the magnitude of differences, interpreted under rehabilitation and sports research guidelines as trivial (<0.2), small (0.2–0.6), moderate (0.6–1.2), large (1.2–2.0), and very large (>2.0) [24]. Precision for all point estimates was indicated using 95% confidence intervals (95% CI) [25].

The examination of the relationship between t30 recorded from the criterion method (photocells) and the examined F-V profile parameters was done with the use of the Kendall’s *τ_b_* correlation coefficient following the results of the Shapiro–Wilk normality test (*p* < 0.05). *τ_b_* values of ±0.00 to ±0.19, ±0.20 to ±0.39, ±0.40 to ±0.59, ±0.60 to ±0.79, and ±0.80 to ±1.00 were interpreted as negligible, weak, moderate, strong, and very strong association, respectively. All analyses were conducted using the Matlab 2023b software (The Mathworks Inc., Natick, MA, USA), with the level of significance set at *a* = 0.05.

## 3. Results

### 3.1. 30 m Sprint Test Performance

The results for t30, as recorded by both the photocell method and the UWB system are displayed in Table 1.

### 3.2. Validity Measures

The alignment between UWB-derived split times and the criterion photocell data varied across the sprint intervals (Table 2).

While the overall 0–30 m sprint exhibited a negligible mean bias (−0.014 s) paired with high absolute agreement (ICC{A,1} = 0.967) and a strong Pearson correlation (*r* = 0.974), the LoA differed by segment. Specifically, the total 0–30 m time displayed the widest LoA (−0.075 to 0.052 s), whereas the 10–20 m segment produced the narrowest (–0.022 to 0.029 s), demonstrating peak consistency during this intermediate phase.

**Figure 1:**
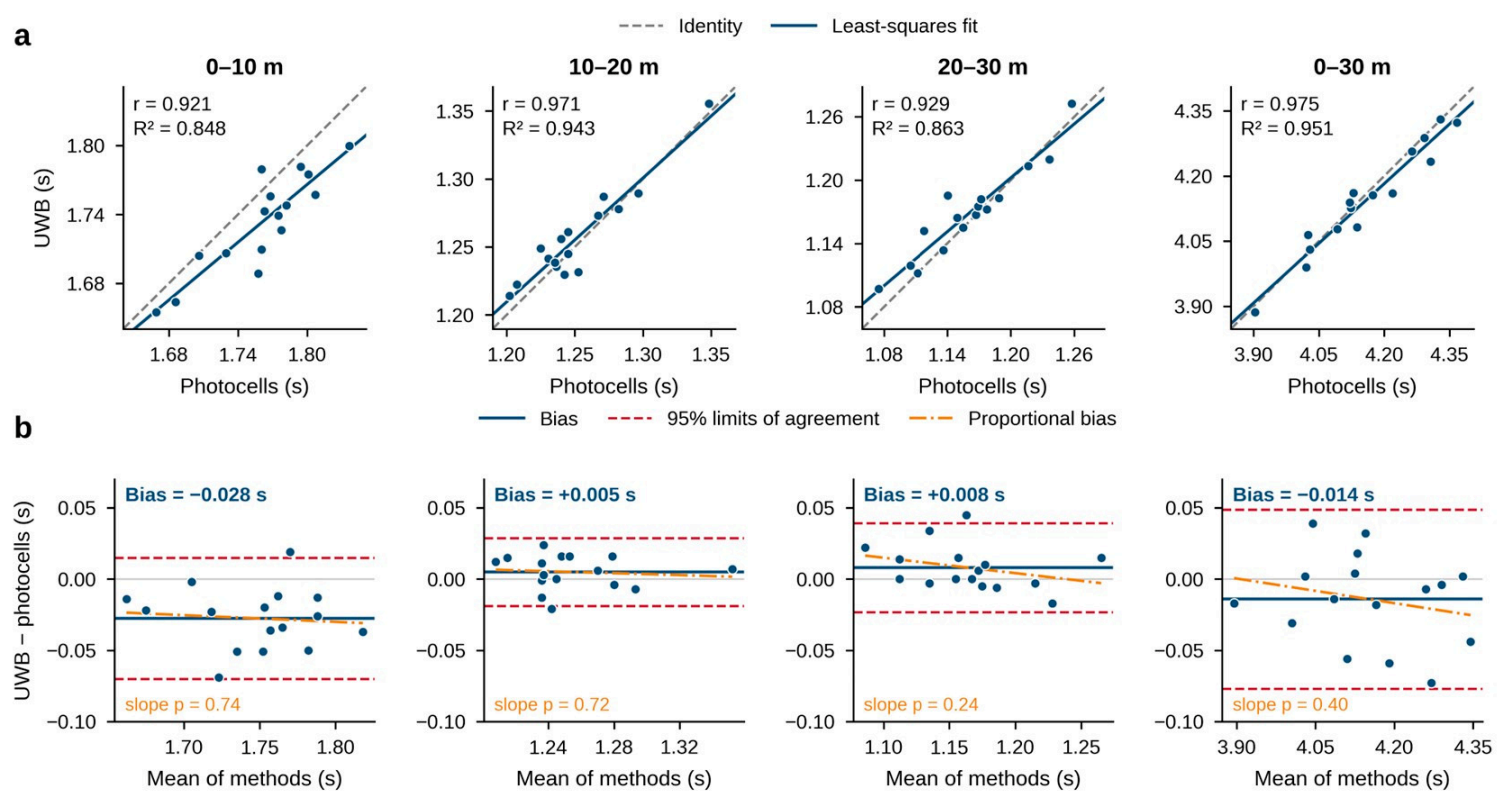
Results of the validity and reliability analysis of the tested K-Power system: (**a**) concurrent validity scatter plots for the Photocells (s) vs. K-Power UWB (s) per sprint interval comparison. Solid line = regression fit; dashed = identity line (perfect agreement). (**b**). Bland–Altman agreement plots for the UWB − Photocells (s) difference vs. mean of methods. Blue line = bias; red dashed line = 95% LoA; orange dash-dotted line = proportional bias trend.

Linear regression analysis of the differences confirmed the absence of proportional bias across all intervals (Figure 1). Furthermore, sensitivity analyses demonstrated that the findings were highly robust and not driven by any individual player, as the maximum absolute change in bias remained negligible across all segments (range: 0.0017–0.0047 s).

### 3.3. Relationship of Sprint Performance with the Force–Velocity Profile Parameters

Average t30 was 4.15 ± 0.12 s, while t10 and t20 were 1.73 ± 0.05 s and 2.98 ± 0.08 s, respectively. Table 3 presents the relationship between t30 and the examined F-V profile parameters. Results revealed that P_MAX_ and V_MAX_ demonstrated significant (*p* < 0.05), negative Kendall’s correlation coefficients with sprint performance. This inverse relationship occurred because lower t30 values indicate faster performance, providing clear evidence that higher power and maximum velocity are linked to lower sprint times. Conversely, no significant relationships were observed between t30 and the other examined parameters (*p* > 0.05).

Regarding the K-Power derived parameters, V_MAX_ was significantly (*p* < 0.05) correlated with V_0_ (*τ_b_* = 0.650, *p* < 0.001), FV_SLOPE_ (*τ_b_* 0.380, *p* = 0.028) and DRF (*τ_b_* = 0.393, *p* = 0.023). No significant relationships were observed between SV_MAX_ and the examined F-V profile parameters (*p* > 0.05).

## 4. Discussion

The primary objective of this study was to assess the concurrent validity and agreement of the K-Power hybrid sensor system against photocell data during short sprints (30 m sprint tests) in young male soccer players. The results indicate that the reported mean biases and LoA widths are stable and not disproportionately influenced by individual outliers. Thus, this investigation showed that the K-Power sensor showed excellent agreement and negligible systematic bias for measuring 30 m sprint performance and its 10 m splits. Based on this finding, the tested sensor system is recommended as a valid practical method for field-based monitoring of linear sprint performance and split times, which can subsequently be used to estimate F-V profile parameters.

Our findings demonstrate high concurrent validity for the hybrid UWB-IMU system in tracking linear sprint times in youth soccer players. Furthermore, this methodological accuracy allowed us to confirm and extend existing research on sprint F-V profiling derived from the analysis of sprint performance metrics. However, when examining individual split intervals, the 0–10 m phase demonstrated the lowest relative agreement between the K-Power system and the timing gates compared to the later splits and the overall 30 m performance. Methodologically, this finding is expected as the initial 10 m represent the most explosive phase of acceleration [26,27], where rapid changes in body posture and foot strike [28] can introduce measurement discrepancies between a body-worn sensor and static photocells or other measurement methods [29]. Despite showing the lowest agreement among the tested intervals, the systematic bias remained small enough to support practical field use. Ensuring acceptable tracking fidelity during this initial 0–10 m window is crucial, as early measurement errors heavily compound when mathematically deriving whole-sprint F-V parameters.

Because measurement tools vary considerably in precision, device choice remains critical [16]. Although timing gates are considered the benchmark tool for measuring sprint speed, results from single-beam and dual-beam setups can yield noticeably different results for individual athletes [30]. Additionally, small timing errors when an athlete triggers the start gate can distort early acceleration data and compromise overall F-V calculations [31]. Alternatives such as GPS and other global navigation satellite systems, radar guns, and low-cost video apps offer acceptable validity for continuous velocity profiling, top speed, and split times. However, GPS systems often exhibit reduced sensitivity during initial acceleration or rapid velocity changes, whereas low-cost video applications (e.g., MySprint) demonstrate reduced validity and greater measurement error when compared directly to radar or GPS devices [16]. Furthermore, derived variables such as the FV_SLOPE_, decrease in the DRF, and early-acceleration metrics frequently show lower reliability across non-continuous tracking systems [32,33,34,35,36]. In contrast, the K-Power system demonstrated strong agreement and minimal systematic bias when compared to a photocell system across both early split intervals and total sprint distance. These results indicate that merging high-frequency IMU motion dynamics with spatial UWB offers a highly practical, field-based solution for profiling the linear speed and mechanical capabilities of young soccer players. This approach overcomes the logistical constraints of traditional devices, such as time-consuming setups and the inability to capture continuous motion [15]. Furthermore, another advantage is that the field-deployable K-Power system can aid coaches for frequent, real-time monitoring, thus enabling them to track and adjust training loads relative to competitive demands [3], besides providing instant feedback to practitioners regarding their sprint performance and the respective F-V profile values.

Our findings show that 30 m sprint performance in U19 soccer players was most strongly associated with V_MAX_, while P_MAX_ demonstrated a significant but moderate association. This is consistent with previous research highlighting that while horizontal force and power production are of importance for sprint performance in young and trained soccer players [9,37,38], the capacity to reach high V_MAX_ plays a primary role in overall performance over a 30 m distance in this cohort. Because both V_MAX_ and P_MAX_ were significantly associated with t30, it seems that 30 m time is not determined by power parameters alone.

Short-sprint acceleration is strongly influenced by horizontal force-production capacity, whereas V_MAX_ is more closely associated with V_0_ and P_MAX_ [9,39]. Given that a 30 m sprint is largely characterized by the acceleration phase, the weak and non-significant relationship between V_0_ and total sprint time observed in this study may reflect the relatively greater importance of acceleration capacity over this distance. However, it is important to note that P_MAX_ and V_MAX_ were both significantly associated with performance, whereas the theoretical variables (F_0_ and V_0_) were not. The absence of a strong link between t30 and F_0_ can be attributed to the fact that F_0_ heavily dictates only the first few explosive steps of the initial acceleration phase. While the 30 m sprint is traditionally framed as an acceleration-dominant test, the data revealed that SV_MAX_ averaged 26.37 ± 2.68 m, indicating that the examined players achieved their actual V_MAX_ very late in the trial, namely at ~88% of the total distance. Therefore, the 30 m sprint test in this cohort captured not only the initial acceleration phase, but also the late-stage transition to near-maximal speed. The lack of association with V_0_ suggests that, while achieving V_MAX_ near the finish line is critical, V_0_ was not fully exploited or manifested within the 30 m distance. Furthermore, although previous research has identified associations between sprint mechanical characteristics and the DRF [36,38], DRF did not reach statistical significance in our specific cohort. Overall, these findings suggest that, in adolescent soccer players, superior performance over a short, game-relevant sprint distance is more closely related to the combination of effective production of horizontal force and mechanical power during acceleration and the capacity to attain high peak velocities near the end of the distance, rather than to theoretical maximal velocity alone [9,40].

While the F-V profile reflects an athlete’s mechanical output, sprint technique also influences the ability to produce high P_MAX_ and F_0_. Faster acceleration is associated with greater forward trunk lean and more effective horizontal force application [41,42]. Longer initial steps and shorter ground contact times are similarly linked to enhanced acceleration performance [43,44]. In addition, optimal trunk and pelvic control may help maintain efficient sprint mechanics, potentially mitigating hamstring injury risk [45,46]. Therefore, future studies should combine field-based sensors, such as the tested K-Power, with three-dimensional motion analysis to examine how sprint technique affects the F-V profile in young athletes.

Assessing these mechanical properties directly in the horizontal plane is critical. A recent systematic review reported that vertical F-V profiling has limited and inconsistent relationships with horizontal sprint performance in soccer [47]. Furthermore, horizontal profiling of young soccer players has identified velocity deficits across different maturational stages [48]. Therefore, horizontal F-V profiling could provide a more sport-specific approach for identifying mechanical deficits and guiding targeted sprint training in adolescent soccer players [47,48]. From a practical standpoint, distinguishing between vertical and horizontal profiles is essential for effective training prescription [49,50]. Traditional resistance training modalities (e.g., squats, deadlifts) primarily target vertical force vectors. While beneficial for general strength, they may not directly address specific deficits in horizontal force production or application during sprinting [51,52]. By using horizontal F-V profiling, coaches and clinicians can precisely identify whether an athlete suffers from a force or velocity deficit during horizontal acceleration [53]. This allows for the selection of vector-specific exercises such as heavy sled towing for force-deficient athletes or assisted/over-speed sprinting for velocity-deficient athletes, thereby optimizing training adaptations and performance transfer to soccer-specific movements [54,55,56,57].

Several limitations should be considered when interpreting the findings of this study. First, the cross-sectional design precludes establishing causal relationships between mechanical F-V parameters and sprint performance; thus, future prospective research is necessary to confirm these predictive associations. Second, the study evaluated a convenience sample of male U19 semi-professional soccer players from a single competitive standard, which limits the generalizability of these results to other age groups, competitive level, or female athletes [7]. Additionally, because the sample size of 18 participants was powered specifically to detect strong correlations, the present study may be underpowered to detect small-to-moderate effects. Consequently, the non-significant, weak-to-moderate associations observed for certain mechanical parameters (e.g., F_0_, FV_SLOPE_) should be interpreted with caution, as a larger sample size may be required to definitively confirm or reject these relationships. Furthermore, the use of this sample deprived the opportunity to check the effect of playing position which is a factor that affects F-V profile parameters [58]. Third, it must be emphasized that the K-Power system was directly validated in this study for sprint times and split intervals rather than F-V profile metrics themselves. The F-V parameters are indirectly estimated from these kinematic data rather than direct in-ground force plate measurements. Although such field-based methods have demonstrated good agreement with force-platform measurements, they remain indirect estimates of the underlying ground-reaction forces [4,59,60]. Furthermore, the analysis focused primarily on linear sprint performance and macroscopic F-V profile parameters, without accounting for other crucial match-related physical demands, such as multi-directional agility, repeated-sprint ability, or individual technical variations. These abilities represent distinct performance qualities and may not be fully captured by linear sprint testing alone [61,62]. Finally, while the concept of the F-V profile analysis is suggested to apply to sprint distances up to 30 m [20], it is challenged for sprint distances of over 10 to 15 m [63]. The latter study suggests that the F-V profile derived from field-based macroscopic data may reflect a mathematical rearrangement of a behavioral velocity-time curve rather than an objective measure of the player’s true neuromuscular capacities. Furthermore, because this model isolates horizontal acceleration, it inherently ignores vertical ground reaction forces, which constitute the largest component of total force production during sprinting. Thus, it is suggested that athletes operate close to their theoretical optimum only within a short 10- to 15 m distance [63]. Beyond this distance, the mathematically derived F-V profile demands an ongoing shift toward velocity capabilities which are difficult to achieve, leading to the argument that key F-V parameters over the 15 m range may represent a theoretical oversimplification rather than a realistic training target [63]. Therefore, our findings should be interpreted with the caveat that while F-V profiling offers clear utility for identifying between-athlete performance variations, its capacity to prescribe highly individualized training adaptations over longer acceleration phases remains a subject of ongoing biomechanical debate.

While the horizontal F-V profiling used in this study provides a comprehensive overview of a player’s bilateral capabilities, it does not account for potential interlimb differences. Because sprinting inherently requires rapid, alternating unilateral force production, inter-limb asymmetries, such as those identified during single-leg lateral jumps, can severely compromise the efficiency of the acceleration phase. Recent evidence suggests that pronounced asymmetry in lower-limb muscle strength and power negatively impacts straight-line running speed and force application in youth and professional athletes [5]. Therefore, addressing this limitation in future research by evaluating the relationship between unilateral force application during lateral jumps, postural control, and the mechanical characteristics of linear sprinting could provide crucial insights into a player’s overall speed and power capabilities.

## 5. Conclusions

In conclusion, the K-Power hybrid UWB-IMU sensor system demonstrates excellent absolute agreement (evidenced by minimal systematic bias and narrow limits of agreement) and high concurrent validity (supported by strong correlations with criterion timing gates) for evaluating linear sprinting capabilities and sprint F-V profiles in young male soccer players. By overcoming the setup constraints and continuous data limitations associated with traditional diagnostic tools, this wearable technology accurately tracks acceleration and instant speed metrics, as well as F-V profile parameters without compromising on-field efficiency. Crucially, the strong relationship found between 30 m sprint performance and both P_MAX_ and V_MAX_ underscores that targeting horizontal power production and velocity execution is essential for speed development in this cohort. Although not all examined F-V parameters demonstrated a clear, statistically significant relationship with sprint performance in this specific cohort, sports practitioners, coaches, and clinicians can still confidently leverage this integrated framework to identify specific mechanical capabilities or velocity deficits. By acknowledging these selective associations, they can design highly tailored, sport-specific training interventions to enhance sprint performance and optimize youth players’ physical fitness.

## Figures and Tables

**Table 1:** Results for the 30 m sprint test performance (t30) and the respective split times at 10 m (t10) and 20 m (t20) for the criterion photocell method and the K-Power system (*n* = 18).

Parameter	Photocell Data (Mean ± SD)	K-Power Data (Mean ± SD)
t10 (s)	1.73 ± 0.05	1.76 ± 0.07
t20 (s)	2.98 ± 0.08	2.98 ± 0.03
t30 (s)	4.15 ± 0.12	4.14 ± 0.24

**Table 2:** Results for the method agreement analyses (*n* = 18).

Split	Bias (s) [95% CI]	LoA (s)	MAE (s)	RMSE (s)	MAPE (%)	*r*	ICC(A,1) [95% CI]	Proportional Bias *R*^2^
0–10 m	−0.028 [−0.043, −0.013]	−0.081 to 0.025	0.030	0.038	1.75	0.921	0.819 [0.399, 0.942]	0.237
10–20 m	0.005 [−0.001, 0.011]	−0.022 to 0.029	0.008	0.012	0.67	0.971	0.955 [0.787, 0.981]	0.277
20–30 m	0.008 [−0.003, 0.018]	−0.031 to 0.046	0.017	0.021	1.48	0.929	0.920 [0.745, 0.964]	0.232
0–30 m	−0.014 [−0.033, 0.004]	−0.075 to 0.052	0.024	0.037	0.57	0.974	0.967 [0.783, 0.993]	0.173

LoA: limits of agreement; MAE: mean absolute error; RMSE: root mean square error; MAPE: mean absolute percentage error; *r*: Pearson’s correlation coefficient; ICC: intraclass correlation coefficient; CI: confidence interval.

**Table 3:** Results for the examined force–velocity profile parameters and their relationship with 30 m sprint performance (*n* = 18).

Parameter	Mean	SD	95% CI	*τ_b_*	*p*
F_0_ (N)	744.92	511.74	472.23–1017.61	−0.254	0.148
V_0_ (m/s)	8.49	0.86	8.03–8.95	−0.051	0.786
P_MAX_ (kW)	1.50	0.79	1.08–1.92	−0.428 *	0.015
FV_SLOPE_	−1.31	1.11	−1.90–−0.71	0.161	0.361
DRF	−10.13	6.20	−13.44–−6.83	0.147	0.402
V_MAX_ (m/s)	8.71	0.51	8.43–8.97	−0.852 *	<0.001
SV_MAX_ (m)	26.37	2.68	24.94–27.81	−0.080	0.648

*: *p* < 0.05; F_0_: maximal theoretical force; V_0_: maximal theoretical velocity; P_MAX_: maximal mechanical power; FV_SLOPE_: slope of the force–velocity profile; DRF: decrease in ratio of force; V_MAX_: maximum velocity; SV_MAX_: distance from the start line where V_MAX_ was achieved CI: confidence interval; *τ_b_*: values of Kendall’s correlation coefficient.

## Data Availability

The data presented in this study are available on request from the corresponding author due to ethical reasons.

## Abbreviations

The following abbreviations are used in this manuscript:

CCC	concordance correlation coefficient
CV	coefficient of variation
DRF	decrease in ratio of force
F-V	force–velocity profile
F_0_	maximal theoretical force
FV_SLOPE_	slope of the force–velocity profile
GPS	global positioning system
ICC	intraclass correlation coefficient
IMU	inertial measurement unit
LoA	limits of agreement
MAE	mean absolute error
MAPE	mean absolute percentage error
MDC	minimal detectable change
P_MAX_	maximal mechanical power
RMSE	root mean square error
SEM	standard error of measurement
SV_MAX_	distance from the start line where V_MAX_ was achieved
t10	time to cover 10 m
t20	time to cover 20 m
t30	time to cover 30 m
TE	typical error
UWB	ultra-wideband
V_0_	maximal theoretical velocity
V_MAX_	maximum velocity
